# High Level Electronic Structure Calculation of Molecular Solid-State NMR Shielding Constants

**DOI:** 10.1021/acs.jctc.1c01095

**Published:** 2022-03-30

**Authors:** Corentin Poidevin, Georgi L. Stoychev, Christoph Riplinger, Alexander A. Auer

**Affiliations:** † Institut des Sciences Chimiques de Rennes, Av. Général Leclerc, 357000 Rennes, France; ‡ Max-Planck-Institut für Kohlenforschung, 45470 Mülheim an der Ruhr, Germany; § FAccTs GmbH, Rolandstr. 67, 50677 Köln, Germany

## Abstract

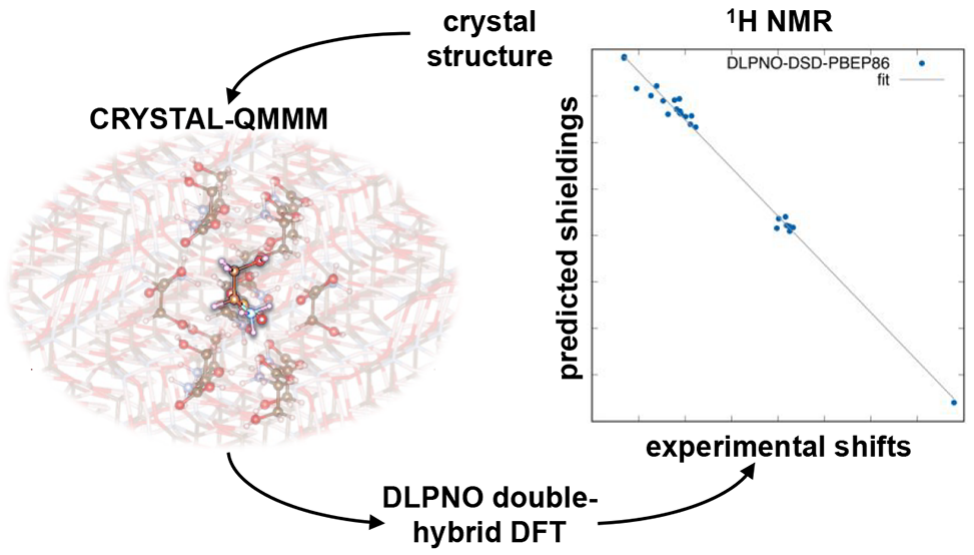

In this work, we present a quantum mechanics/molecular mechanics (QM/MM) approach for the computation of solid-state nuclear magnetic resonance (SS-NMR) shielding constants (SCs) for molecular crystals. Besides applying standard-DFT functionals like GGAs (PBE), meta-GGAs (TPSS), and hybrids (B3LYP), we apply a double-hybrid (DSD-PBEP86) functional as well as MP2, using the domain-based local pair natural orbital (DLPNO) formalism, to calculate the NMR SCs of six amino acid crystals. All the electronic structure methods used exhibit good correlation of the NMR shieldings with respect to experimental chemical shifts for both ^1^H and ^13^C. We also find that local electronic structure is much more important than the long-range electrostatic effects for these systems, implying that cluster approaches using all-electron/Gaussian basis set methods might offer great potential for predictive computations of solid-state NMR parameters for organic solids.

## 1 Introduction

Besides X-ray diffraction, solid-state nuclear magnetic resonance (SS-NMR) spectroscopy has become a powerful way to study molecular crystal structures. SS-NMR can be used for powder or amorphous samples and yields information about the local environment of NMR active nuclei. However, the structure cannot be resolved solely from experimental NMR data. Thus, SS-NMR results are often combined with plane wave density functional theory (DFT) electronic structure calculations with gauge-including projector augmented plane waves (GIPAW) to validate or refine structural information.^1−3^ While periodic DFT has the advantage of simulating the crystal environment, calculations of NMR properties are in the vast majority of the cases limited to the use of (meta-)GGA functionals which have been shown to produce NMR shielding constants (SCs) of limited accuracy.^4−7^ Typically, the SCs are highly sensitive to the description of the electronic structure near the nucleus. As a consequence, in molecular calculations that aim at high accuracy, it is common to use special basis sets that include Gaussians with high exponents, and post-Hartree–Fock (HF) calculations require the inclusion of core correlation. In contrast to this, periodic boundary calculations commonly apply effective core potentials even for light atoms, and the GIPAW approach for NMR calculations combines the reconstruction of the core region using the projector augmented wave technique in combination with gauge-including orbitals to approximate a core electron density that yields satisfactory accuracy in DFT applications.

In parallel to the plane wave approach, quantum mechanics/molecular mechanics (QM/MM) models of molecular crystals have also been developed for the calculation of NMR SCs. This approach mainly consists of focusing on a central QM region, composed of an asymmetric unit surrounded by a few other asymmetric units, which is embedded in MM point charges. Here the aim is to concentrate the computational effort on obtaining the best possible local electronic environment for the central asymmetric unit by using higher level methods for the electronic structure as well as atom-centered basis sets. However, this implies that the QM region has to be of limited size, as higher-level electronic structure methods can become extremely expensive computationally. To circumvent this issue, over the last 10 years Beran et al. have developed a fragment-based method allowing large QM regions to be tackled at affordable computational costs.^8−11^ On the other hand, cluster approaches with or without MM point charges have also been used.^11−14^ In the cluster approach, the whole QM region is included in the NMR calculation while only considering the NMR SCs of the central molecule.

As the size of the QM system in the QM/MM approach can become rather big if one wants to include all the adjacent molecules to the central asymmetric unit, the literature only reports results using (meta-)GGA or hybrid functionals for the calculation of SCs for SS-NMR. However, double-hybrid functionals such as DSD-PBEP86 or B2PLYP and post-HF methods like MP2 have been shown to lead to a great improvement of accuracy for the calculation of NMR SCs on small molecules, when compared with precise but computationally demanding methods like coupled cluster theory.^15,16,5,17^ Recently, Dračínský et al. presented a study on the molecular crystals of six amino acids using MP2 or CCSD corrections on PBE GIPAW results.^18^ These corrections were obtained by calculating the isolated single amino acid molecules at a higher level of theory. However, the main improvement of their results was found when they included vibrational effects via the use of path-integral molecular dynamics (PIMD).

A recent study in our group focused on using a QM/MM approach to compute the NMR SCs for ionic solids.^15^ In this work it was found that the size of the QM region is more important than the way the embedding was done. Furthermore, MP2 and DH functionals were found to lead to a significant improvement over standard DFT for the calculation of SCs, giving more consistent results for all tested nuclei, in agreement with what has been discussed in the framework of fragmentation or composite methods to compute NMR parameters.^19,20^ However, these improvements come at a price, as methods that include post-SCF correlation are both inherently more expensive and require larger basis sets to converge toward the complete basis set (CBS) limit, compared to other DFT approaches. Kaupp and co-workers, for example, have shown that, for NMR shielding calculations, local hybrid functionals fall between conventional functionals and DHs in terms of both accuracy and computational cost.^21,22^

Recently, local correlation methods for the calculation of NMR SCs at MP2 and DH functional levels have been developed and implemented.^23−26^ This allows for the use of high-level electronic structure methods with large QM/MM clusters without drastic increase of computational effort. Thus, as a proof of concept and first benchmark, we have decided to study the same amino acid molecular crystals that Dračínský et al. have studied and use their experimental results.^18^ However, we use nonperiodic methods with embedding as this allows us to apply methods developed for molecular systems. Namely, we use the domain-based local pair natural orbital (DLPNO) approximation as this has been shown to provide high accuracy and allows sufficiently large systems to be treated in the QM region.^26−28^ Ultimately, we wish to investigate whether this approach yields better accuracy than GIPAW in a black-box way for practical applications. Our main focus here is on the ^1^H NMR shieldings, as these are the most common experimentally but computationally challenging in SS-NMR.

## 2 Structures and Method

### 2.1 Studied Systems

**Figure 1:**
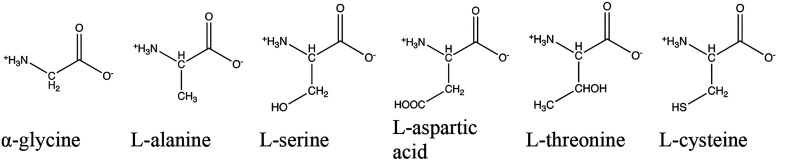
The six studied amino acids.

As in the work of Dračínský et al., the structures of the 6 amino acids (namely, α-glycine, l-alanine, l-serine, l-aspartic acid, l-cysteine, and l-threonine) were obtained from the Cambridge Structural Database (CSD refcodes: GLYCIN29, LTHREO01, LASPRT, LCYSTN21, LSERIN01, and LALNIN12, Figure 1).^29^ These amino acids display a large variety of H environments, i.e., C–H, S–H, N–H, and O–H, which in the latter two cases form hydrogen bonds with neighboring oxygen from carboxylates.

### 2.2 Embedded Cluster Approach and Computational Details

**Figure 2:**
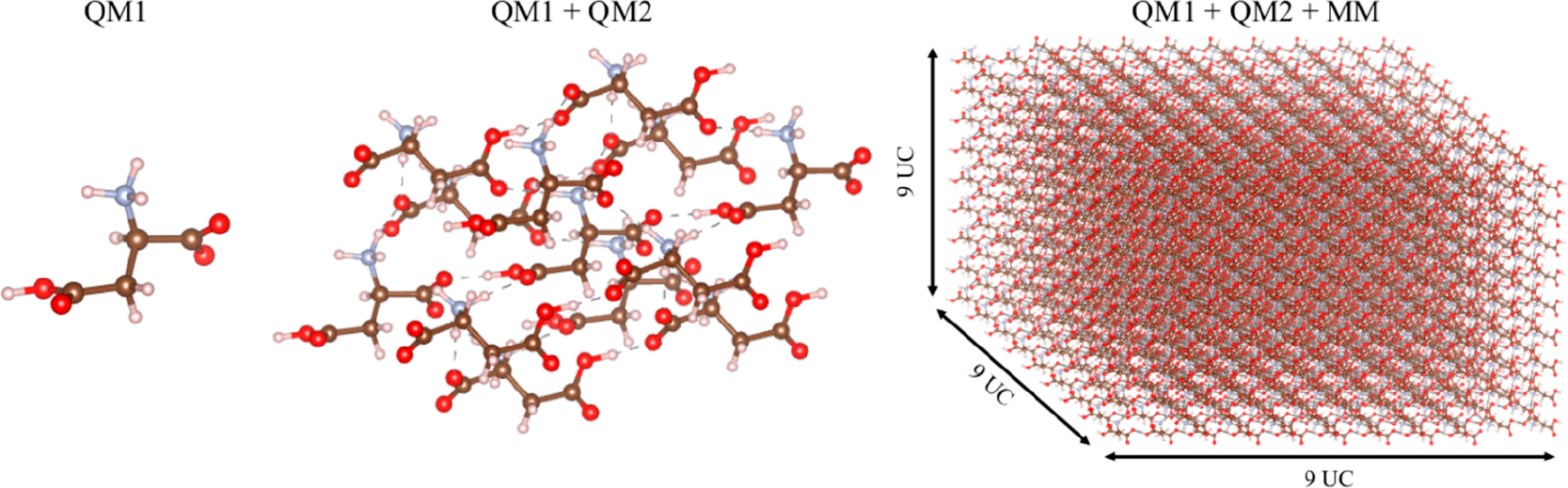
QM1 corresponds to the molecule (asymmetric unit) on which the NMR properties are calculated. QM2 corresponds to the first shell of the asymmetric units around QM1 (i.e., with at least one atom within 2.5 to 3.5 Å depending on the system). The MM region corresponds to the duplication of 9 × 9 × 9 unit cells around QM1 and is composed of self-consistently optimized point charges equal to the CHELPG charges calculated for the QM1 atoms.

Our cluster model for the calculation of NMR chemical shifts consists of (1) an asymmetric unit (QM1) of the central unit cell for which the NMR chemical shifts are calculated, (2) the first shell of asymmetric units (QM2) around QM1 (i.e., molecules with at least one atom within 2.5 to 3.5 Å depending on the system), and (3) a point charge field corresponding to the duplication of 9 × 9 × 9 unit cells around the central cell (MM) as depicted in Figure 2. In order to include the first shell of molecules around QM1, a total of 13 molecules were used for the QM systems (QM1+QM2) in all cases with the exception of α-glycine, where 14 had to be used.

All calculations were carried out using a development version of the ORCA 5.0 program package.^30,31^ As mentioned above, the NMR properties are only reported for the QM1 molecules in the presence of the QM2 molecules and the point charges using gauge including atomic orbitals (GIAOs). The electronic structures of QM1 and QM2 are calculated using the same method. In this study we used four classes of functionals, GGA (PBE), meta-GGA (TPSS), hybrid (B3LYP), and DH (DSD-PBEP86) as well as MP2.^32−36^ In all cases, the RI (PBE and TPSS) or RIJCOSX approximations are used, and the atom-pairwise dispersion correction with the Becke-Johnson damping scheme (D3BJ) was used for the DFT calculations.^37−39^ In order to reduce the computational cost, a smaller basis set is often used for the QM2 molecules (def2-TZVP or pcSseg-2 instead of pcSseg-3).^40,41^ In all cases the DefGrid3 grid settings were used. Note that, in this work, we have applied the ad hoc gauge-invariant approach for the kinetic energy density (τ) terms in meta-GGA functionals, which is the default in ORCA 5. Recent work has demonstrated that a more rigorous treatment is provided by the Dobson ansatz for τ,^42−44^ although the differences for some functionals, such as TPSS, are small.^21^ We have included data calculated with TPSS and the Dobson ansatz in the Supporting Information (Table S9), and indeed, the results are very similar.

The charges of the MM region are optimized by converging them to those of the atomic (CHELPG) charges of the central asymmetric unit (QM1) through successive SCF procedures down to a threshold of 0.01.^12,45^ For this purpose the atomic charges of the QM atoms are first computed without the surrounding point charges. These atomic charges are then mapped onto the equivalent MM atoms (the super cell consists of repeating molecules). The atomic charges are then iteratively computed in the field of the surrounding MM charges, until convergence is achieved. The property calculation is then carried out using the converged atomic charges for the MM atoms. Note that we have not included relativistic effects in the computation of the NMR chemical shifts, as we mostly focus on light atoms. However, in the presence of heavier nuclei, this might lead to deviations—in the case of sulfur, up to 0.5 ppm for ^1^H SCs.^21,46,47^

The experimental crystal structures are used as the initial structure for the construction of our models. Then, we optimize the positions of the hydrogen atoms of the QM1 molecule at the PBE-D3BJ/def2-SVP level in the presence of the fixed QM2+MM embedding. Once optimized, the whole QM1+QM2+MM is rebuilt using the new positions of the hydrogen atoms. This structure is then used for the calculation of the SCs.

Due to the cost of calculating such large systems at the DH or MP2 level, the DLPNO approximation is used in both cases, combined with a multilevel approach, as described in ref (48). MP2 correlation contributions from electron pairs involving at least one occupied orbital localized to the QM1 fragment were treated at the “NormalPNO” level and the rest at the “LoosePNO” level.^26−28^ This approximation saves computational time and is expected to have a negligible effect on the calculated shieldings.

Molecular dynamic (MD) simulations were also done to estimate vibrational effects (see Section 3.5). These MD simulations were done at the GFN2-xTB level at 300 K with a time step of 1 fs.^49,50^ Non-hydrogen atoms were held fixed. The total run length was 40 ps. After 1 ps of equilibration, 100 snapshots (evenly separated by 0.3 ps) were used for single point calculations. The successive snapshot calculations (see Section 3.5) were performed directly on the GFN2-xTB geometries, as these have been reported to yield reasonable structures also for hydrogen bonded systems.^50^

The correlation between experimental shifts and computed shieldings was analyzed using a linear regression fit (σ_*calc*_ = *a*δ_*exp*_ + *b*), for which the slope (*a*), intercept (*b*), and coefficient of determination *R*^2^ are reported. The predicted shifts (δ_*calc*_ = (σ_*calc*_ – *b*)/*a*) from the fit were used to calculate the mean absolute error (MAE), maximum absolute error (MaxAE), and standard deviation of errors (SDE), see Section 1 in the SI for details. Chemical shifts were also calculated with respect to a computed reference value for the shielding in tetramethylsilane (TMS). These were also used to calculate MAE, MaxAE, SDE, and mean signed error (MSE). The structure of TMS was optimized at the B3LYP-D3BJ/def2-TZVP level using a conductor-like polarizable continuum model (CPCM) for water.^51,52^ The experimental reference is usually an aqueous solution of sodium 3-(trimethylsilyl)propanesulfonate (DSS); however, the ^1^H chemical shift difference between TMS and DSS is less than 0.02 ppm and thus largely immaterial for the discussion here.^53^ Note that while in SSNMR it is common to investigate linear regressions when comparing theory and experiment, choosing a suitable reference or schemes like the MSTD approach^54^ might be advantageous for practical applications as it provides the desired cancellation of systematic errors and is not dependent on knowledge of a set of experimental values.

## 3 Results and Discussion

In the following sections, we go through the various influences of the approximations made in embedded cluster electronic structure calculations on ^1^H and ^13^C chemical shifts. While we will discuss the influence of basis set and electronic structure methods, the main focus lies on the question whether embedded cluster calculations allow the properties of molecular methods to be carried over to the description of NMR parameters for molecules in the solid. This includes also additional schemes like molecular higher order correlation corrections or approximate treatments of vibrational effects.

### 3.1 ^1^H NMR Chemical Shifts—Cluster Embedding

To study the influence of the long-range electrostatic environment on the calculated NMR shielding, the effect of the point charges (MM part) has to be assessed. First, we find that the converged MM charges do not fluctuate significantly with the level of calculation (see Table S1 in the Supporting Information). Second, we also calculated the NMR SCs of the six systems at the B3LYP level without including the point charges, as can be seen in the second line of Table 1 (“B3LYP (QM1/QM2)”). The obtained slope is the same as with the MM embedding. On the other hand, the *R*^2^, the MAE, and the MaxAE are found to be slightly worse. As an illustration we also calculated the NMR SCs of the six molecules without QM2 and MM (see the line “B3LYP (QM1)” of Table 1) for which the correlation with the experimental chemical shifts is, as expected, very poor (*R*^2^ = 0.58). Finally, we performed calculations in which the QM2 region was instead treated using point charges (labeled “B3LYP (QM1/MM)”), which is a commonly used treatment in the literature. Interestingly, the *R*^2^ coefficient for this fit is much better at 0.93, but the slope and intercept of the fit differ significantly from the ideal −1 and the TMS reference shielding, respectively. Thus, at the QM1/MM level of embedding, reasonably quantitative results can be obtained, but only if the systematic errors are reduced, e.g., using a linear regression model. This shows that even though the MM embedding is an important part of the model, the most important aspect is the local chemical environment and electronic structure.

**Table 1:** Correlation Parameters between Experimental ^1^H Shifts and the Calculated NMR Shieldings Calculated with B3LYP and Different Levels of Embedding^a^^,^^b^

	slope	intercept	*R*^2^	MAE	MaxAE^c^		SDE
B3LYP	–1.03	31.14	0.9915	0.21	0.68	SH(cys)	0.30
B3LYP (QM1/QM2)	–1.03	31.09	0.9869	0.26	0.89	SH(cys)	0.37
B3LYP (QM1/MM)	–0.57	29.91	0.9313	1.06	4.85	COOH(asp)	1.46
B3LYP (QM1)	–0.38	29.44	0.5806	1.81	7.90	OH(thr)	2.72

a Intercept, MAE, MaxAE, and SDE are given in ppm, while the slope and *R*^2^ are unitless.

b See text for embedding scheme details.

c Maximum error and the corresponding hydrogen atom.

### 3.2 ^1^H NMR Chemical Shifts–Basis Set, COSX, and DLPNO Errors

The basis set is less likely to be a significant source of error here, as can be seen when comparing results from Table 2 and Table S3 in the Supporting Information: when using a smaller basis set (i.e., psSseg-2 for QM1 and def2-SVP for QM2) only slightly different results are obtained. Additionally, for MP2, we also evaluated the error induced by using the RIJCOSX approximation compared to RIJONX. As can be seen from Table 3, the RIJCOSX approximation does not lead to a significant increase in the error of the NMR SCs. The multilevel scheme for the DLPNO approximations does deteriorate the results slightly, compared to the full NormalPNO level, but not to an extent that would change any of our conclusions.

**Table 2:** Correlation Parameters between Experimental ^1^H Shifts and the Calculated NMR Shieldings Corresponding to the Values from Table 4^a^

	slope	intercept	*R*^2^	MAE	MaxAE^b^		SDE
PBE	–1.00	30.73	0.9911	0.22	0.72	SH(cys)	0.30
TPSS	–1.00	31.13	0.9917	0.22	0.65	SH(cys)	0.29
B3LYP	–1.03	31.14	0.9915	0.21	0.68	SH(cys)	0.30
DLPNO-DSD-PBEP86	–1.04	31.13	0.9907	0.22	0.79	SH(cys)	0.31
DLPNO-MP2	–1.04	31.00	0.9909	0.22	0.78	SH(cys)	0.31
PBE(GIPAW)^c^	–1.10	30.75	0.9864	0.22	1.48	SH(cys)	0.38

a Intercept, MAE, MaxAE, and SDE are given in ppm, while the slope and *R*^2^ are unitless.

b Maximum error and the corresponding hydrogen atom.

c GIPAW values from the work of Dračínský et al.^18^

**Table 3:** Correlation Parameters between Experimental ^1^H Shifts and the Calculated NMR Shieldings Corresponding to the Values Calculated at the DLPNO-MP2 Level Using pcSseg-3 (See also Table 4), pcSseg-2 (See also Table S2), and pcSseg-2 with NormalPNO Together with either RIJONX or RIJCOSX Approximations^a^

DLPNO-MP2 settings	slope	intercept	*R*^2^	MAE	MaxAE^b^		SDE
Multilevel, pcSseg-3, RIJCOSX	–1.04	31.00	0.9909	0.22	0.78	SH(cys)	0.31
Multilevel, pcSseg-2, RIJCOSX	–1.05	30.89	0.9875	0.26	0.89	SH(cys)	0.36
NormalPNO, pcSseg-2, RIJONX	–1.04	31.09	0.9910	0.22	0.73	SH(cys)	0.31
NormalPNO, pcSseg-2, RIJCOSX	–1.04	31.10	0.9910	0.22	0.73	SH(cys)	0.31

a Intercept, MAE, MaxAE, and SDE are given in ppm, while the slope and *R*^2^ are unitless.

b Maximum error and the corresponding hydrogen atom.

### 3.3 ^1^H NMR Chemical Shifts–Level of Theory, DFT vs DH-DFT, and GIAO vs GIPAW

**Figure 3:**
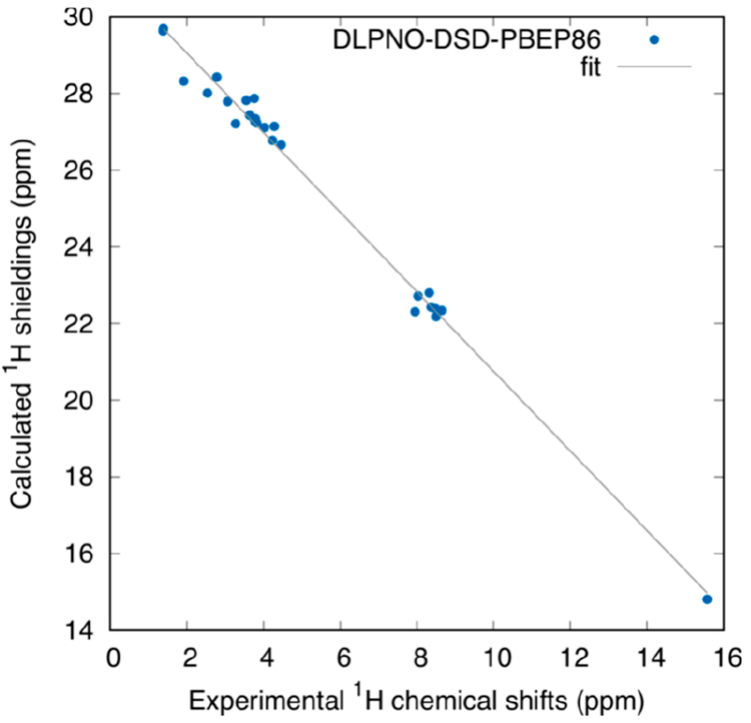
^1^H shieldings (ppm) vs experimental ^1^H chemical shifts and the linear fit calculated at the DLPNO-DSD-PBEP86 level using the values from Table 4.

The calculated ^1^H NMR shieldings using the scheme described in Section 2.2 are reported in Table 4 along with the experimental ^1^H chemical shifts from the work of Dračínský et al.^18^ The linear fit parameters and statistical analysis are presented in Table 2, and an illustration of the fitted data is shown in Figure 3 (see Figure S1 in the SI for the rest of the fits). Surprisingly, all the electronic structure methods used (GGA (PBE), Meta-GGA (TPSS), hybrid (B3LYP), double-hybrid (DSD-PBEP86), and MP2) exhibit good correlation with the experiment in terms of slope, close to the ideal −1.0, *R*^2^ coefficients of determination around 0.99, MAEs of 0.22 ppm, and maximum errors of better than 0.8 ppm. Note that Dračínský et al. reports slopes around −1.1, MAEs of 0.22–0.26 ppm, and maximum errors of 1.49–1.76 ppm for PBE(GIPAW) values and values using corrections to the electronic structure from molecular calculations.

**Table 4:** Experimental ^1^H Chemical Shifts and the Corresponding Calculated NMR Shieldings in ppm

	hydrogen	δ_exp_^a^	PBE^b^	TPSS^b^	B3LYP^b^	DLPNO-DSD-PBEP86^c^	DLPNO-MP2^c^
l-alanine	H-α	3.82	26.91	27.28	27.23	27.23	27.09
	NH_3_	8.5	22.15	22.57	22.25	22.18	22.04
	H-β	1.38	29.43	29.81	29.67	29.63	29.52
α-glycine	NH_3_	8.48	22.35	22.81	22.46	22.39	22.25
	H-α1	4.23	26.53	26.91	26.78	26.78	26.67
	H-α2	3.06	27.51	27.90	27.79	27.79	27.69
l-serine	H-α	3.64	27.05	27.41	27.42	27.43	27.32
	H-β1	3.75	27.03	27.45	27.38	27.34	27.21
	H-β2	4.46	26.28	26.75	26.67	26.66	26.51
	NH_3_	8.37	22.37	22.84	22.50	22.43	22.30
	OH	3.79	27.49	27.89	27.55	27.34	27.10
l-aspartic acid	COOH	15.57	15.00	15.45	14.96	14.81	14.58
	H-β1	3.27	26.86	27.33	27.21	27.21	27.09
	H-β2	2.54	27.73	28.13	28.00	28.01	27.81
	NH_3_	8.32	22.70	23.11	22.82	22.80	22.63
	H-α	3.76	27.56	27.95	27.90	27.87	27.69
l-cysteine	H-β1	3.55	27.44	27.90	27.80	27.82	27.69
	H-β2	2.78	28.11	28.50	28.50	28.43	28.23
	H-α	4.28	26.74	27.13	27.10	27.14	27.00
	NH_3_	8.65	22.24	22.70	22.36	22.34	22.20
	SH	1.92	28.09	28.57	28.46	28.32	28.19
l-threonine	NH_3_	8.03	22.62	23.07	22.77	22.72	22.53
	H-α	4.02	26.79	27.17	27.11	27.11	26.93
	H-β	3.78	26.86	27.31	27.29	27.26	27.08
	OH	7.95	22.39	22.84	22.44	22.31	22.17
	H-γ	1.39	29.53	29.92	29.78	29.69	29.59

a Experimental values from ref (18).

b Values calculated using the scheme described in Section 2.2 with pcSseg-3 and pcSseg-2 basis sets for QM1 and QM2, respectively.

c pcSseg-3 and def2-TZVP basis sets for QM1 and QM2, respectively, were used, and NormalPNO and LoosePNO settings using the fragment scheme presented in Section 2.2 were used.

**Figure 4:**
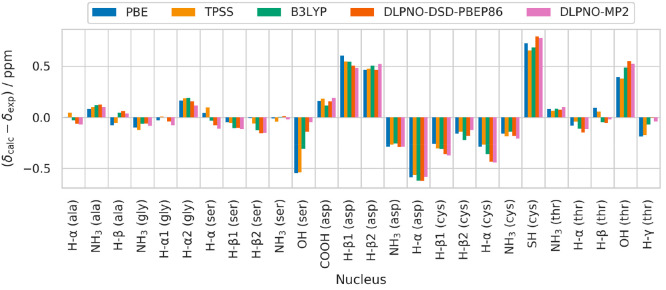
Errors between experimental ^1^H shifts and those calculated with each method from the data in Table 4 using the linear fit parameters in Table 2.

As in the study of Dračínský et al., we observe the largest deviations for S–H of l-cysteine, but they are still smaller than when using PBE GIPAW. Two positions for the S–H are found in the crystal structure of l-cysteine, either S–H···S or S–H···O.^55−57^ Thus, we computed both structures and took an average of the NMR shielding values. However, both structures lead to similar shieldings for all ^1^H, including the S–H (±0.15 ppm), and therefore do not improve the overall correlation, which is also what was observed by Dračínský et al. Other hydrogen atoms show significant deviations from the fit, namely, the O–H from l-serine and l-threonine and the H-α, H-β_1_, and H-β_2_ of l-aspartic acid. Note that for several protons the deviations are mostly independent of the method used (see Figure 4). This suggests they are likely due to issues other than the electronic structure, like the reference geometries or vibrational effects. Relativistic spin–orbit (SO) effects are unlikely to be the source of these errors, although they can explain the large error for the S–H proton. Results calculated using the Δσ_SO_ corrections from ref (18) are presented in the SI. It is apparent that none of the other nuclei (for which Δσ_SO_ is below 0.1 ppm) benefit from the correction, while the fit parameters and other statistical quantities are only marginally improved.

Overall, the smallest deviations are obtained with the TPSS functional, while results using perturbative correlation corrections (DLPNO-DSD-PBEP86 and DLPNO-MP2) are slightly worse than those using other standard DFT functionals. This is in contrast to what has been found in previous studies for heavier nuclei, where DH-DFT often is an improvement over GGA or hybrid functional DFT,^26^ but there are indications in the literature that error for hydrogen shifts can be quite different than for heavier nuclei, particularly when comparing to experimental, rather than ab initio, reference data.^58^ It is also worth noting that, even using the DLPNO approach, MP2 and DH calculations take about 10 times longer than PBE or TPSS calculations and about 5 times longer than the B3LYP ones (see Figure S5 in the SI).

Statistical parameters for the chemical shifts calculated with respect to TMS are shown in Table S5 and errors for individual nuclei in Figure S3 in the SI. If a reference compound is chosen to evaluate and compare relative chemical shifts, rather than doing a linear fit, there are significant systematic deviations in these results, as can be expected. This can be due the very different accuracies of the calculated shieldings for the amino acids and the reference system, for example, caused by the different electronic structure and treatment of the environment. The reference shielding at the PBE level is 31.35 ppm, which differs from the “optimal” value of 30.73 ppm, given by the intercept of the linear fit, by 0.62 ppm. This accounts for the MSE and MAE and is almost half of the MaxAE. This demonstrates that the linear fit removes most of the systematic error and gives a better estimate of the “best case” performance of each method. Thus, we mostly focus on the linear fit results in the rest of the discussion.

### 3.4 ^1^H NMR Chemical Shifts—Higher Level Corrections from Molecular Calculations

In the study of Dračínský et al. of the same amino acids, they used the difference of shieldings between the single molecule calculated at the PBE level and those calculated at a higher level of theory (e.g., PBE0, MP2, or CCSD) as corrections to their PBE-GIPAW results. In their study, this correction alone did not lead to an improvement of the correlation with respect to the experimental shifts for ^1^H NMR. In order to assess whether such procedures could be efficiently used in combination with our cluster approach, we calculated the NMR shieldings of the single molecules at the PBE and DSD-PBEP86 levels. This allows us to evaluate the correction that should be added to our QM/MM PBE results by comparison with the full embedded cluster results obtained at the DSD-PBEP86 of theory. As can be seen in Table 5, this scheme, at least with a DSD-PBEP86 correction, does not lead to an improvement of our results, but rather to their deterioration. While the slope and *R*^2^ values for the full embedded cluster approach for PBE and DSD-PBEP86 are −1.00 and 0.9911 and −1.04 and 0.9907, respectively, the corrected PBE values are −1.05 and 0.9849. We would argue here that this indicates nontransferability of correlation effects from the molecule to the solid due to the effect of the local crystal structure (intermolecular interaction) on the electronic structure. Inspection of the rows labeled “QM1” as well as Table 1 reveals that the molecular results show very large deviations, so it is reasonable that a correction obtained without embedding will yield very different results than the higher level calculation with full embedding. Note that our findings are in agreement with what Dračínský et al. report. In their work, corrections obtained from molecular MP2 and CCSD calculations actually increase the deviations from experiment.

**Table 5:** Correlation Parameters between Experimental ^1^H Shifts and the Calculated NMR Shieldings Corresponding to the Values from Table 4 for PBE and DLPNO-DSD-PBEP86 and PBE Values from the Cluster Scheme plus the Difference between the Calculated Values at the PBE and DSD-PBEP86 Levels of the Single Molecules (QM1)^a^

	slope	intercept	*R*^2^	MAE	MaxAE^b^		SDE
PBE	–1.00	30.73	0.9911	0.22	0.72	SH(cys)	0.30
DLPNO-DSD-PBEP86	–1.04	31.13	0.9907	0.22	0.79	SH(cys)	0.31
PBE (QM1)	–0.35	29.07	0.5108	2.17	8.94	OH(thr)	3.13
DSD-PBEP86 (QM1)	–0.39	29.41	0.6351	1.59	6.93	OH(thr)	2.43
PBE + Δσ_DSD-PBEP86_	–1.05	31.06	0.9849	0.28	1.12	SH(cys)	0.40

a Intercept, MAE, MaxAE, and SDE are given in ppm, while the slope and *R*^2^ are unitless.

b Maximum error and the corresponding hydrogen atom.

### 3.5 ^1^H NMR Chemical Shifts–Assessment of Vibrational Corrections

In an attempt to further improve our results, we performed molecular dynamics simulations, allowing only the hydrogen atoms of the QM1 molecule to move. We then took the average of NMR shielding calculations from 100 snapshots for each system at the PBE level of theory. This approach has been chosen as it does not require extensive full dynamics simulations or the expensive computation of anharmonic effects but still allows the impact of the vibrational correction to the calculated SCs to be assessed (see PBE-MD in Table 6). Note that zero-point and anharmonic vibrational effects on NMR shieldings should typically be captured using a quantum treatment like perturbation theory.^16,59,60^ However, several studies suggest that MD+snapshot calculations allow for fairly robust estimates of dynamic effects with high efficiency.^61−64^

**Table 6:** Correlation Parameters between Experimental ^1^H Shifts and the Calculated NMR Shieldings Corresponding to the Mean of 100 Calculations at the PBE Level at Snapshot Geometries from the Hydrogen MD Simulation (Denoted “PBE-MD”)^a^

	slope	intercept	*R*^2^	MAE	MaxAE		SDE
PBE-MD	–1.04	30.82	0.9901	0.23	0.88	OH(ser)	0.32
B3LYP + DIFF	–1.06	31.35	0.9929	0.20	0.68	OH(ser)	0.27
DLPNO-MP2 + DIFF	–1.07	31.33	0.9936	0.20	0.61	H-β1(asp)	0.26
DLPNO-DSD-PBEP86 + DIFF	–1.07	31.21	0.9926	0.21	0.60	H-β1(asp)	0.28

a B3LYP, DLPNO-MP2, and DLPNO-DSD-PBEP86 values correspond to the values from Table 4 plus the difference between the “PBE-MD” values and those from the static calculations at the PBE level.

The difference between the NMR shieldings calculated for the rigid system (Table 1) and the average of the 100 snapshots is then used as a correction (DIFF in Table 6). This correction, calculated at the PBE level, is added to results of the previously calculated NMR shieldings using other levels of theory as can be seen in Table 6. One can see a small improvement of the MAE and the MaxAE in all cases but for the PBE results, which might hint as some level of error compensation. However, in all cases the slope deviates more from the ideal −1 compared to the static results.

### 3.6 ^13^C NMR Chemical Shifts—Similarities and Differences for Heavier Nuclei

**Figure 5:**
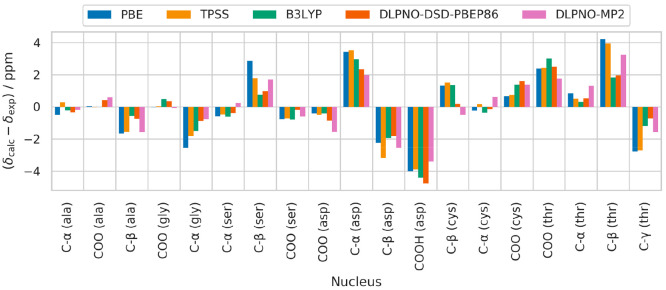
Errors between experimental ^13^C shifts and those calculated with each method from the data in Table 7 using the linear fit parameters in Table 8.

In the following, we will discuss the calculated carbon NMR shieldings. The results, using the scheme described in Section 2.2, are reported in Table 7 along with the experimental carbon chemical shifts from the work of Dračínský et al.^18^ The correlation between experimental shifts and computed shieldings is again obtained with a linear fit, and the fit parameters and statistical analysis are presented in Table 8. A good correlation between the experimental shifts and the calculated shieldings is found for all tested computational methods. In all cases, the slope is found to be very close to the ideal −1. The MAEs are between 1.1 and 1.7 ppm, and the MaxAEs are between 4 and 5 ppm, which is acceptable for molecular solids. Here we find that both the MAE and the SDE values improve as the level of theory increases. While the number of values is relatively small, and one has to be careful not to overanalyze the trends observed here, it appears that, especially for the heavier nuclei, post-HF correlation effects improve the results, which is in line with what is known from molecular systems. Our values are slightly worse than those obtained by Dračínský et al. using PBE and GIPAW. This could be due to the fact that, contrary to their study, the position of the carbon atoms was not relaxed in our cluster calculations as relaxing the whole geometry of the crystal is less straightforward in cluster-based calculations. A look at the error of the chemical shifts calculated for individual nuclei in Figure 5 also shows indications for this. As for the ^1^H case, large errors for some nuclei are observed, especially in l-aspartic acid, which are mostly independent of the method used. In the future, we might consider combining structures calculated with a periodic approach and compute the NMR SCs with our cluster approach. Note that just as for the hydrogen SCs, molecular DSD-PBEP86 corrections on embedded PBE results do not lead to an improvement of the agreement between computed values and the experiment. Note that, for heavier elements, work by Dračínský et al. reports that such a correction can be beneficial.^65^ However, here the authors corrected GIPAW GGA results with molecular hybrid functional results, and one could also speculate that the observed improvement is rather due to correcting the GIPAW error than the functional error. In our correction scheme, however, we correct embedded Gaussian basis set models using higher level nonembedded Gaussian basis set results, so that mostly the effect of transferability of correlation effects is observed.

**Table 7:** Experimental ^13^C Chemical Shifts and the Corresponding Calculated NMR Shieldings in ppm

	carbon	δ_exp_^a^	PBE^b^	TPSS^b^	B3LYP^b^	DLPNO-DSD-PBEP86^b^^,^^c^	DLPNO-MP2^c^
l-alanine	C-α	50.92	122.75	128.71	122.55	134.94	137.95
	COO	177.71	–3.28	4.56	–8.58	7.33	12.23
	C-β	20.36	154.14	160.50	154.46	165.91	169.45
α-glycine	COO	176.25	–1.78	5.92	–7.56	8.88	14.35
	C-α	43.58	132.05	137.97	131.45	142.82	145.77
l-serine	C-α	55.69	118.11	124.76	118.04	130.21	132.84
	C-β	62.86	107.61	115.51	109.22	121.68	124.33
	COO	175.05	0.13	7.86	–5.01	10.60	16.04
l-aspartic acid	COO	175.91	–1.05	6.80	–6.28	10.43	16.14
	C-α	53.78	116.04	122.71	116.32	129.40	133.00
	C-β	37.77	137.49	145.01	137.91	149.58	153.27
	COOH	174.66	3.73	11.35	–0.88	15.57	19.18
l-cysteine	C-β	28.09	143.55	149.91	144.50	157.26	160.78
	C-α	56.01	117.45	123.82	117.45	129.65	132.15
	COO	173.37	0.39	8.07	–5.52	10.50	15.74
l-threonine	COO	172.06	–0.02	7.70	–5.85	10.90	16.65
	C-α	61.25	111.19	118.34	111.34	123.75	126.31
	C-β	66.93	102.23	109.40	103.91	116.63	118.81
	C-γ	20.48	155.13	161.52	155.00	165.77	169.34

a Experimental values from ref (18).

b Values calculated using the scheme described in Section 2.2 with pcSseg-3 and pcSseg-2 basis sets for QM1 and QM2, respectively.

c pcSseg-3 and def2-TZVP basis sets for QM1 and QM2, respectively, were used, and NormalPNO and LoosePNO settings using the fragment scheme presented in Section 2.2 were used.

**Table 8:** Correlation Parameters between Experimental ^13^C Shifts and the Calculated NMR Shieldings Corresponding to the Values from Table 7 and Various Approximate Schemes (See Text)^a^

	slope	intercept	*R*^2^	MAE	MaxAE^b^		SDE
PBE	–0.99	172.66	0.9989	1.66	4.22	C-β(thr)	2.18
TPSS	–0.98	178.96	0.9990	1.57	3.94	COOH(asp)	2.10
B3LYP	–1.03	174.90	0.9993	1.27	4.41	COOH(asp)	1.74
DLPNO-DSD-PBEP86	–1.00	185.54	0.9994	1.14	4.76	COOH(asp)	1.64
DLPNO-MP2	–0.99	187.96	0.9993	1.35	3.40	COOH(asp)	1.70
B3LYP (QM1/QM2)	–1.03	174.54	0.9990	1.56	4.99	COOH(asp)	2.05
B3LYP (QM1/MM)	–1.01	173.96	0.9987	1.88	4.61	C-β(ala)	2.35
B3LYP (QM1)	–0.95	171.02	0.9977	2.29	7.09	C-β(thr)	3.14
PBE (QM1)	–0.92	170.76	0.9956	3.03	10.29	C-β(thr)	4.31
DSD-PBEP86 (QM1)	–0.92	181.88	0.9979	2.31	6.41	C-β(thr)	2.96
PBE + Δσ_DSD-PBEP86_	–0.99	183.78	0.9984	2.07	5.09	C-α(asp)	2.58
PBE(GIPAW)^c^	–1.02	173.05	0.9995	1.09	3.21	C-β(thr)	1.49

a Intercept, MAE, MaxAE, and SDE are given in ppm, while the slope and *R*^2^ are unitless.

b Maximum error and the corresponding carbon atom.

c GIPAW values from the work of Dračínský et al.^18^

## 4 Conclusions

For ^1^H NMR chemical shifts of molecular crystals, we find that an embedded cluster approach yields robust and accurate results compared to experimental values. While a sufficiently large cluster with explicit neighboring molecules is essential, MM embedding beyond that only slightly improves the results. In agreement with previous studies with experimental reference data, we find that functionals like PBE and TPSS yield fairly accurate results ,while DH-DFT only yields an improvement for ^13^C NMR chemical shifts. Standard basis sets like Jensen’s pcSseg-2 and -3 yield sufficiently converged results, and an estimate of vibrational corrections based on a simple MD sampling approach shows that these effects are small even for light nuclei like ^1^H.

An interesting finding for ^1^H NMR chemical shifts is that results from the cluster approach are actually superior to the GIPAW results discussed by Dračínský et al., while this is not entirely true for the ^13^C NMR chemical shifts. This raises the question whether this is due to effects of the partially optimized structures or if the reconstruction of core orbitals in the GIPAW scheme introduces larger errors for the light nuclei. Here, more work comparing appropriately embedded GIAO and periodic GIPAW results for identical systems is needed.

Using the cluster approach in combination with local correlation approximations allows us to test transferability corrections like the one proposed in the work by Dračínský et al., where molecular calculations at the Coupled Cluster level have been used to derive post-DFT corrections to the DFT-GIPAW results. Comparing pure PBE embedded cluster results with results obtained by combining embedded cluster PBE with molecular DSD-PBEP86 corrections shows that the obtained accuracy is notably worse than the actual embedded cluster DSD-PBEP86 values for both ^1^H and ^13^C. This hints at a nonadditivity for details of the electronic structure in the molecule and its interaction with the local environment. Hence, from our results it seems advisable to choose the best possible level of theory for an embedded cluster model rather than investing resources in highly accurate molecular calculations to derive corrections.

When new local correlation methods like DLPNO-based Coupled Cluster can be combined with the embedded cluster GIAO scheme presented in this work, this might provide a path to highly accurate calculations with predictive power even for systems as complex as molecular crystals.

## Supporting Information Available

Data analysis, CHELPG charges as a function of the method, ^1^H isotropic NMR SCs calculated with looser parameters and correlation fits, linear fit calculated using settings from Table 4, ^1^H chemical shifts with spin-orbit corrections, ^1^H chemical shifts using the calculated reference shielding in TMS, ^13^C isotropic NMR SCs calculated with looser parameters and correlation fits, ^13^C chemical shifts using the calculated reference shielding in TMS, shieldings calculated with TPSS and the Dobson treatment of the kinetic energy density, and timings for the calculation needed for the NMR CS calculations using the settings from Table 4 in the main text (PDF)
