# Supplementary material for: High Level Electronic Structure Calculation of Molecular Solid-State NMR Shielding Constants
Source: J Chem Theory Comput. 2022 Mar 30;18(4):2408–17. doi: 10.1021/acs.jctc.1c01095 (PMC9009078; doi:10.1021/acs.jctc.1c01095)
Supplement: Supplementary file 1 — ct1c01095_si_001.pdf [file ct1c01095_si_001.pdf]

Supplementary Information to:

High level electronic structure calculation of molecular solid-state NMR shielding constants

Corentin Poidevin, Georgi L. Stoychev, Christoph Riplinger and Alexander A. Auer

1. CHELPG charges as a function of the method..2
Table S1..2
2. ^{1}H Isotropic NMR SCs calculated with looser parameters and correlation fits......................2
Table S2..2
Table S3..3
3. Linear fit calculated using values from Table 4..4
Figure S1..4
4. ^{1}H chemical shifts with spin-orbit corrections..5
Table S4..5
Figure S2..5
5. ^{1}H chemical shifts using the calculated reference shielding in TMS.....................................6
Figure S3..6
Table S5..6
6. ^{13}C isotropic NMR SCs calculated with looser parameters and correlation fits.....................7
Table S6..7
Table S7..8
7. ^{13}C chemical shifts using the calculated reference shielding in TMS.....................................8
Table S8..8
Figure S4..8
8. Shieldings calculated with TPSS and the Dobson treatment of the kinetic energy density…9
Table S9..9
9. Timings for the calculation needed for the NMR CSs calculations using the setting from Table 4 and 7 in the main text..10
Figure S5..10

1. CHELPG charges as a function of the method

Table S1. α-glycine CHELPG converged charged (MM) as a function of the electronic calculation method using pcSseg-3 and def2-TZVP basis sets for QM1 and QM2, respectively. MAD: mean absolute deviation.

	PBE	TPSS	B3LYP	DLPNO-DSD-PBEP86	DLPNO-MP2	MAD
O	-0.712	-0.725	-0.742	-0.784	-0.778	0.026
O	-0.715	-0.726	-0.743	-0.764	-0.745	0.014
N	-0.444	-0.451	-0.465	-0.509	-0.570	0.041
C(COO)	0.672	0.686	0.699	0.720	0.689	0.013
C(α)	-0.002	0.018	0.023	0.004	-0.058	0.022
H(NH_3)	0.350	0.358	0.359	0.376	0.440	0.025
H(NH_3)	0.275	0.277	0.283	0.302	0.327	0.017
H(NH_3)	0.376	0.379	0.397	0.450	0.515	0.047
H(α1)	0.096	0.094	0.097	0.123	0.188	0.029
H(α2)	0.021	0.016	0.016	0.028	0.045	0.009

2. ^{1}H Isotropic NMR SCs calculated with looser parameters and correlation fits

The isotropic NMR SCs were also calculated with looser parameters to evaluate their dependence on the size of the basis set and PNO threshold. Tables S2 and S3 report the calculated ^{1}H NMR SCs and their correlation to the experimental chemical shifts, respectively. The calculations were carried out using pcSseg-2 and def2-SVP basis sets for QM1 and QM2, respectively, for PBE, TPSS and B3LYP and DLPNO-DSD-PBEP86 and pcSseg-3 and def2-SVP basis sets for QM1 and QM2, respectively, for MP2. Additionally, LoosePNO settings were used for DLPNO-DSD-PBEP86 and DLPNO-MP2.

Table S2. Experimental ^{1}H chemical shifts and the corresponding calculated NMR SCs in ppm. [a] Experimental values from the work of Dračínsky et al. (ref. 18 in main text). [b] Values calculated using the scheme described in section 2.2 with pcSseg-2 and def2-SVP basis sets for QM1 and QM2, respectively. [c] LoosePNO settings were used here. [d] Values calculated using the scheme described in section 2.2 with pcSseg-3 and def2-SVP basis sets for QM1 and QM2, respectively.

	Hydrogen	δ_{exp}[a]	PBE[b]	TPSS[b]	B3LYP[b]	DLPNO-DSD-PBEP86[b,c]	DLPNO-MP2[d,c]
L-alanine	H-α	3.82	27.01	27.41	27.33	27.37	27.07
	NH_3	8.5	22.17	22.61	22.28	22.21	21.72
	H-β	1.38	29.47	29.87	29.71	29.68	29.40
α-glycine	NH_3	8.48	22.34	22.79	22.46	22.46	22.19
	H-α1	4.23	26.46	26.86	26.71	26.79	26.61

	H-α2	3.06	27.50	27.88	27.77	27.82	27.57
L-serine	H-α	3.64	27.00	27.39	27.36	27.45	27.14
	H-β1	3.75	26.91	27.30	27.25	27.23	26.96
	H-β2	4.46	26.21	26.63	26.59	26.60	26.18
	NH_3	8.37	22.26	22.70	22.38	22.35	21.95
	OH	3.79	27.52	27.89	27.59	27.47	27.03
L-aspartic acid	COOH	15.57	15.01	15.45	14.96	14.83	14.41
	H-β1	3.27	26.80	27.23	27.15	27.15	26.84
	H-β2	2.54	27.70	28.07	27.97	28.03	27.79
	NH_3	8.32	22.78	23.20	22.90	22.86	22.59
	H-α	3.76	27.65	28.03	28.00	28.04	27.87
L-cysteine	H-β1	3.55	27.62	28.07	27.96	28.00	27.78
	H-β2	2.78	27.85	28.28	28.22	28.24	27.69
	H-α	4.28	26.26	26.65	26.56	26.59	26.48
	NH_3	8.65	22.16	22.60	22.26	22.25	22.18
	SH	1.92	28.24	28.66	28.59	28.47	28.23
L-threonine	NH_3	8.03	22.73	23.18	22.88	22.94	22.48
	H-α	4.02	26.78	27.18	27.09	27.13	26.79
	H-β	3.78	26.87	27.28	27.30	27.38	27.12
	OH	7.95	22.44	22.86	22.48	22.39	21.76
	H-γ	1.39	29.55	29.95	29.79	29.76	29.49

As can be seen in Table S3, the use of a smaller basis set has minimal effect on the correlation between the calculated NMR SCs and the experimental chemical shifts.

Table S3. Correlation parameters between experimental ^1H shifts and the calculated NMR SCs corresponding to the values from Table S2. [a] Maximum Error and the corresponding hydrogen atom.

	Slope	Intercept	R^2	MAE[a]	MaxAE[a]		SDE
PBE	-1.00	30.71	0.9904	0.23	0.70	SH(L-cys)	0.32
TPSS	-0.99	31.10	0.9905	0.23	0.68	SH(L-cys)	0.31
B3LYP	-1.03	31.10	0.9913	0.21	0.74	SH(L-cys)	0.30
DLPNO-DSD-PBEP86	-1.04	31.14	0.9905	0.22	0.77	SH(L-cys)	0.31
DLPNO-MP2	-1.05	30.89	0.9875	0.26	0.89	SH(L-cys)	0.36

3. Linear fit calculated using values from Table 4

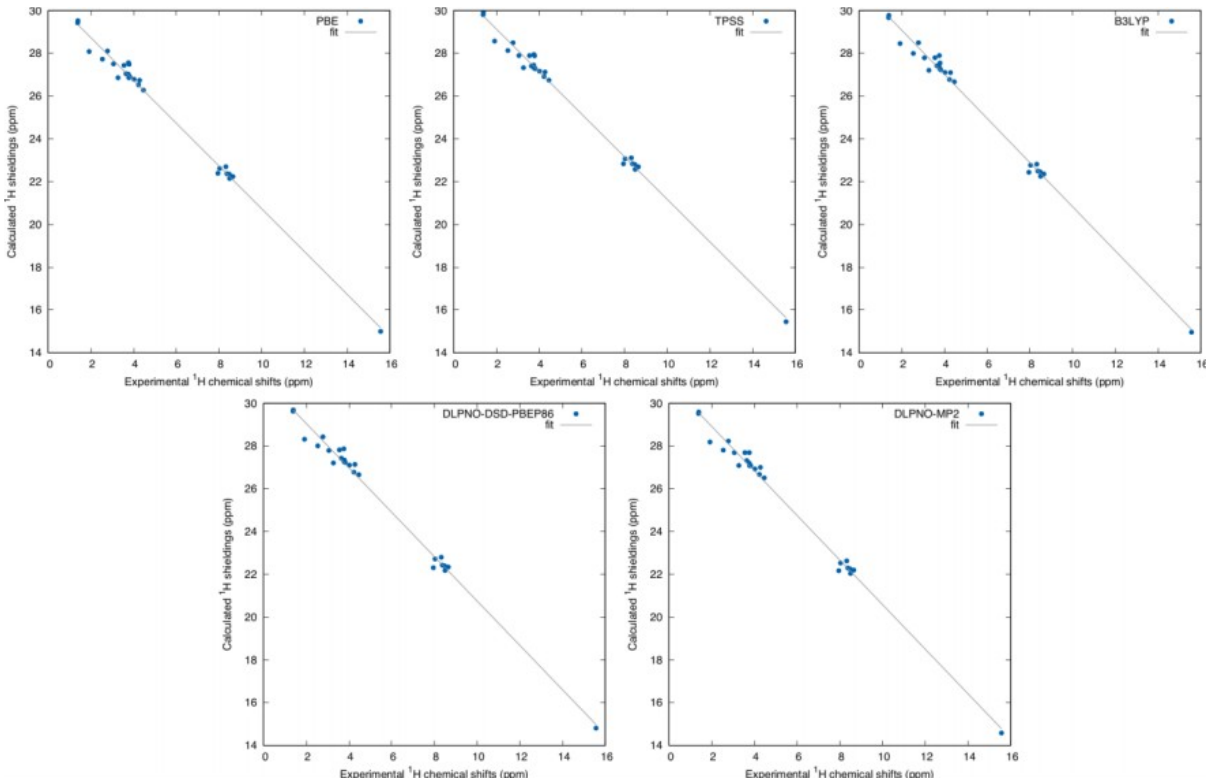

Figure S1. ¹H shieldings (ppm) vs experimental ¹H chemical shifts and the linear fit calculated at each level using the values from Table 4.

4. ^{1}H chemical shifts with spin-orbit corrections

Table S4. Correlation parameters between experimental ^{1}H shifts and the calculated NMR shieldings corresponding to the values from Table S2, corrected by adding the $\Delta\sigma_{SO}$ values from the work of Dračínsky et al.[18] Intercept, MAE, MaxAE, and SDE are given in ppm, while the slope and R^2 are unitless. [a] Maximum Error and the corresponding hydrogen atom.

	Slope	Intercept	R^2	MAE[a]	MaxAE[a]		SDE
PBE + $\Delta\sigma_{SO}$	-1.00	30.79	0.9932	0.19	0.61	H-β1(asp)	0.26
TPSS + $\Delta\sigma_{SO}$	-1.00	31.18	0.9936	0.18	0.57	OH(ser)	0.26
B3LYP + $\Delta\sigma_{SO}$	-1.03	31.19	0.9936	0.18	0.57	H-α(asp)	0.26
DLPNO-DSD-PBEP86 + $\Delta\sigma_{SO}$	-1.04	31.18	0.9934	0.19	0.58	H-α(asp)	0.26
DLPNO-MP2 + $\Delta\sigma_{SO}$	-1.04	31.05	0.9936	0.18	0.56	H-β2(asp)	0.26

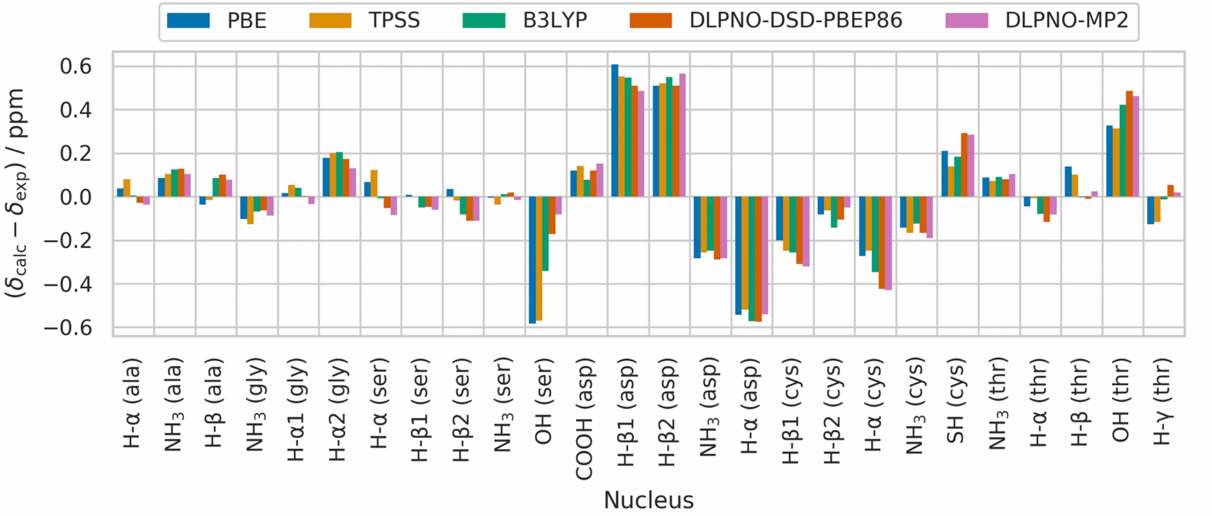

Figure S2. Errors between experimental ^{1}H shifts and those calculated with SO correction using the linear fit parameters in Table S4.

5. [1]H chemical shifts using the calculated reference shielding in TMS

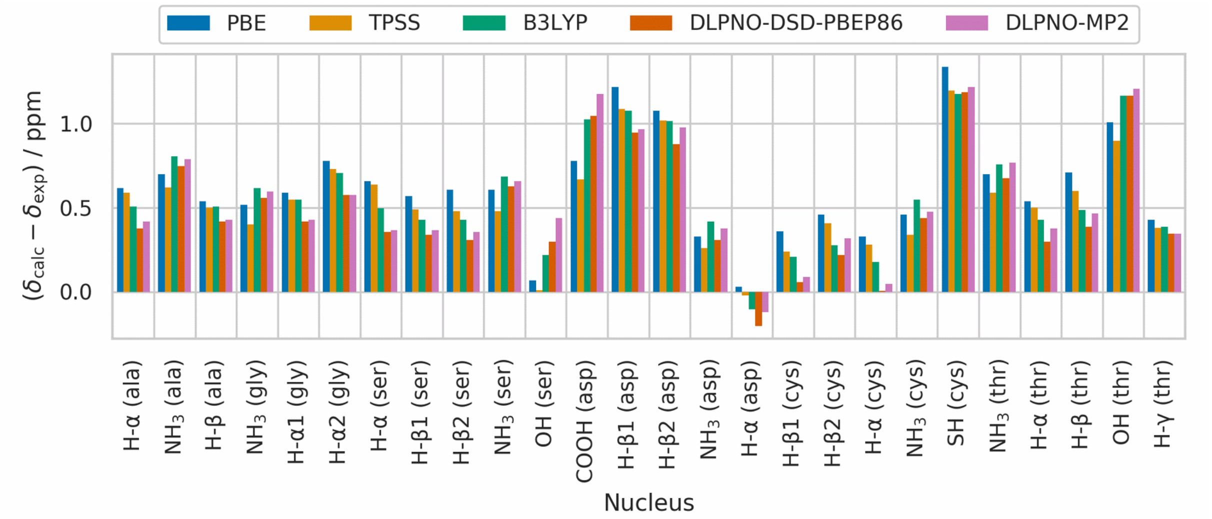

Figure S3. Errors between experimental ^{1}H shifts and those calculated with each method from the data in Table 4 using TMS as a reference compound. Reference shieldings are given in the caption of Table S5.

Table S5. Errors in the computed ^{1}H chemical shifts using the calculated reference shielding in TMS. All values are given in ppm. The reference ^{1}H shieldings for each method are: 31.35 (PBE), 31.69 (TPSS), 31.56 (B3LYP), 31.43 (DLPNO-DSD-PBEP86), and 31.33 (DLPNO-MP2) ppm. [a] Maximum Error and the corresponding hydrogen atom.

	MSE	MAE	MaxAE[a]		SDE
PBE	0.62	0.62	1.34	SH(cys)	0.30
TPSS	0.54	0.54	1.20	SH(cys)	0.29
B3LYP	0.58	0.58	1.18	SH(cys)	0.32
DLPNO-DSD-PBEP86	0.49	0.51	1.19	SH(cys)	0.34
DLPNO-MP2	0.54	0.55	1.22	SH(cys)	0.35
B3LYP (QM1/QM2)	0.59	0.61	1.42	SH(cys)	0.39
B3LYP (QM1/MM)	-0.55	1.06	4.85	COOH(asp)	1.46
B3LYP (QM1)	-1.09	1.68	7.51	COOH(asp)	2.25

6. ^{13}C isotropic NMR SCs calculated with looser parameters and correlation fits

Tables S5 and S6 report the calculated ^{13}C NMR SCs and their correlation to the experimental chemical shifts, respectively. The calculations were carried out using pcSseg-2 and def2-SVP basis sets for QM1 and QM2, respectively, for PBE, TPSS and B3LYP and DLPNO-DSD-PBEP86 and pcSseg-3 and def2-SVP basis sets for QM1 and QM2, respectively, for MP2. Additionally, LoosePNO settings were used for DLPNO-DSD-PBEP86 and DLPNO-MP2.

Table S6 Experimental ^{13}C chemical shifts and the corresponding calculated NMR SCs in ppm. [a] Experimental values from the work of Dračínsky et al.[18] *[b] Values calculated using the scheme described in section 2.2 with pcSseg-2 and def2-SVP basis sets for QM1 and QM2, respectively. [c] LoosePNO settings were used here. [d] Values calculated using the scheme described in section 2.2 with pcSseg-3 and def2-SVP basis sets for QM1 and QM2, respectively.*

	Carbon	δ_{exp}[a]	PBE[b]	TPSS[b]	B3LYP[b]	DLPNO-DSD-PBEP86[b,c]	DLPNO-MP2[d,c]
L-alanine	C-α	50.92	123.30	129.04	123.24	136.91	138.19
	COO	177.71	-2.93	4.76	-8.02	9.44	12.03
	C-β	20.36	154.63	160.90	155.08	167.52	169.66
α-glycine	COO	176.25	-1.37	5.98	-6.97	10.72	13.59
	C-α	43.58	132.34	138.20	131.89	144.71	146.36
L-serine	C-α	55.69	117.65	124.77	117.65	131.24	133.62
	C-β	62.86	106.22	115.17	106.22	121.76	124.82
	COO	175.05	3.33	8.08	3.33	15.63	15.85
L-aspartic acid	COO	175.91	-0.83	6.83	-5.84	11.89	14.42
	C-α	53.78	116.41	122.77	116.80	130.80	132.12
	C-β	37.77	138.06	145.03	138.57	151.31	153.09
	COOH	174.66	4.25	11.70	-0.17	17.47	20.54
L-cysteine	C-β	28.09	144.03	150.00	144.91	158.98	161.38
	C-α	56.01	117.06	123.28	117.15	130.86	131.64
	COO	173.37	2.78	10.10	-2.78	14.62	17.21
L-threonine	COO	172.06	0.10	7.64	-5.52	12.51	15.68
	C-α	61.25	111.70	118.45	111.98	125.63	126.48
	C-β	66.93	102.70	109.62	104.53	118.59	119.62
	C-γ	20.48	155.87	161.59	155.81	167.62	169.69

Table S7 Correlation parameters between experimental ^{13}C shifts and the calculated NMR SCs corresponding to the values from Table S2. [a] Maximum Error and the corresponding carbon atom.

	Slope	Intercept	R^2	MAE	MaxAE[a]		SDE
PBE	-0.98	172.63	0.9985	2.00	4.61	C-β(L-ser)	2.54
TPSS	-0.98	178.92	0.9989	1.67	3.88	COOH(L-asp)	2.18
B3LYP	-1.02	174.53	0.9982	1.99	7.19	COOH(L-asp)	2.72
DLPNO-DSD-PBEP86	-0.99	186.76	0.9992	1.39	4.17	COO(L-ser)	1.88
DLPNO-MP2	-0.99	188.31	0.9991	1.42	4.90	COOH(L-asp)	1.90

7. [13]C chemical shifts using the calculated reference shielding in TMS

Table S8. Errors in the computed ^{13}C chemical shifts using the calculated reference shielding in TMS. All values are given in ppm. The reference ^{13}C shieldings for each method are: 182.01 (PBE), 188.62 (TPSS), 181.66 (B3LYP), 192.05 (DLPNO-DSD-PBEP86), and 196.35 (DLPNO-MP2) ppm. [a] Maximum Error and the corresponding carbon atom.

	MSE	MAE	MaxAE[a]		SDE
PBE	8.40	8.40	12.85	C-β(thr)	2.26
TPSS	7.92	7.92	12.29	C-β(thr)	2.39
B3LYP	9.81	9.81	15.45	COOH(thr)	2.77
DLPNO-DSD-PBEP86	6.55	6.55	9.09	COOH(thr)	1.64
DLPNO-MP2	7.03	7.03	10.61	C-β(thr)	1.92
B3LYP (QM1/QM2)	9.79	9.79	15.90	COOH(thr)	2.81
B3LYP (QM1/MM)	8.93	8.93	12.83	C-α(asp)	2.53
B3LYP (QM1)	5.91	6.21	14.00	C-β(thr)	4.43

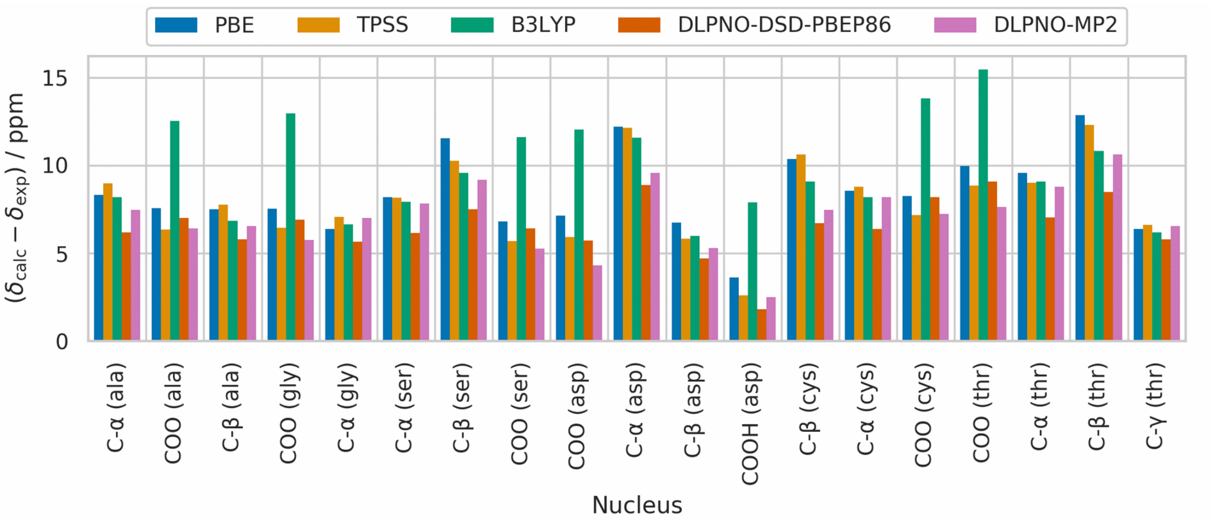

Figure S4. Errors between experimental ^{13}C shifts and those calculated with each method from the data in Table 7 using TMS as a reference compound.

8. Shieldings calculated with TPSS and the Dobson treatment of the kinetic energy density

Table S9. Calculated shieldings at the TPSS level using the scheme described in section 2.2 with pcSseg-3 and pcSseg-2 basis sets for QM1 and QM2, respectively, and either the ad-hoc gauge invariant or Dobson treatment for the kinetic energy density. Statistical error analysis for the shifts calculated using either a linear fit or TMS as reference, with respect to experimental data, is given below.

	Hydrogen	Ad-hoc	Dobson	Carbon	Ad-hoc	Dobson
L-alanine	H-α	27.28	27.27	C-α	128.71	126.04
	NH_3	22.57	22.44	COO	4.56	1.07
	H-β	29.81	29.73	C-β	160.50	157.20
α-glycine	NH_3	22.81	22.64	COO	5.92	2.30
	H-α1	26.91	26.87	C-α	137.97	134.98
	H-α2	27.90	27.84			
L-serine	H-α	27.41	27.42	C-α	124.76	121.83
	H-β1	27.45	27.38	C-β	115.51	112.12
	H-β2	26.75	26.65	COO	7.86	4.35
	NH_3	22.84	22.65			
	OH	27.89	27.73			
L-aspartic acid	COOH	15.45	15.30	COO	6.80	3.33
	H-β1	27.33	27.23	C-α	122.71	119.89
	H-β2	28.13	28.03	C-β	145.01	141.43
	NH_3	23.11	22.98	COOH	11.35	7.99
	H-α	27.95	27.91			
L-cysteine	H-β1	27.90	28.00	C-β	149.91	146.93
	H-β2	28.50	28.14	C-α	123.82	120.10
	H-α	27.13	26.56	COO	8.07	6.37
	NH_3	22.70	22.35			
	SH	28.57	28.45			
L-threonine	NH_3	23.07	22.91	COO	7.70	4.19
	H-α	27.17	27.11	C-α	118.34	115.38
	H-β	27.31	27.27	C-β	109.40	106.70
	OH	22.84	22.64	C-γ	161.52	157.71
	H-γ	29.92	29.82			

Continued on next page…

… Continued from previous page		Hydrogen		Carbon	
		Ad-hoc	Dobson	Ad-hoc	Dobson
Linear fit	Slope	-1.00	-1.01	-0.98	-0.98
	Intercept	31.13	31.05	178.96	175.80
	R^2	0.9917	0.9912	0.9990	0.9991
	MAE	0.22	0.21	1.57	1.52
	MaxAE	0.65	0.67	3.94	3.74
	SDE	0.29	0.30	2.05	1.91
Versus TMS	σ_{ref}	31.69	31.62	188.62	184.43
	MSE	0.54	0.60	7.92	6.92
	MAE	0.54	0.60	7.92	6.92
	MaxAE	1.20	1.25	12.29	10.80
	SDE	0.29	0.30	2.39	2.26

9. Timings for the calculation needed for the NMR CSs calculations using the setting from Table 4 and 7 in the main text.

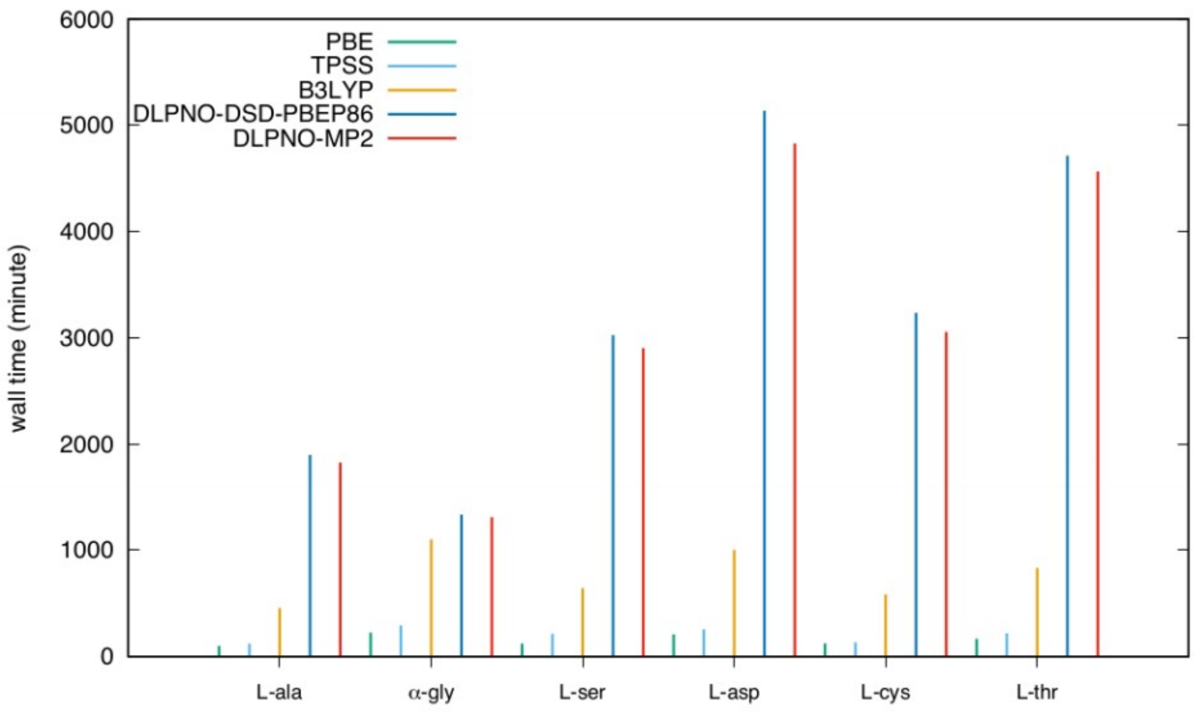

Figure S5. Total wall clock times (on Intel Xeon E5-2687Wv4 (3 GHz CPU) using 12 processors) for the NMR shielding calculations corresponding to the settings from Table 5 as a function of the electronic structure method.
